# The relationship between insulin resistance and fibroblast growth factor 23 in patients with non-diabetic pre-dialysis chronic kidney disease: a cross-sectional study

**DOI:** 10.1590/1516-3180.2024.0103.03072024

**Published:** 2025-01-27

**Authors:** Beyza Algul Durak, Melahat Coban

**Affiliations:** I Department of Nephrology, Ankara Bilkent City Hospital, Ankara, Turkey; II Associate Professor, Department of Nephrology, Ankara Bilkent City Hospital, Ankara, Turkey

**Keywords:** Fibroblast growth factor 23, Creatinine, Glomerular filtration rate, Pre-dialysis chronic kidney disease, Soluble klotho, Homeostasis model assessment of insulin resistance

## Abstract

**BACKGROUND:**

Insulin resistance often occurs in patients with chronic kidney disease (CKD) owing to mineral and bone metabolism disorders. Fibroblast growth factor (FGF)-23 and soluble klotho (s-KL) play crucial roles in linking CKD with mineral and bone metabolism.

**OBJECTIVE:**

This study aimed to examine the relationship between insulin resistance and FGF-23 and s-KL in patients with non-diabetic pre-dialysis patients with CKD.

**DESIGN AND SETTING:**

This research was conducted in the Ankara Bilkent City Hospital Nephrology Clinic. Ankara,Turkey.

**METHODS:**

This study included 133 male and 150 female patients with pre-dialysis CKD. The patients were compared with 80 healthy individuals. FGF-23 and s-KL levels were determined using enzyme-linked immunosorbent assay kits. The homeostasis model assessment of insulin resistance (HOMA-IR) was used to determine insulin resistance.

**RESULTS:**

Creatinine, urine protein/creatinine ratio (UPCR), log_10_ FGF-23, log _10_ s-KL, and HOMA-IR were notably higher, while glomerular filtration rate was notably lower, in patients than in healthy individuals. Stage 5 CKD, log_10_ FGF-23, creatinine, and UPCR were significantly higher in patients with HOMA-IR > 3.06 compared to those with HOMA-IR ≤ 3.06. No difference was observed in s-KL levels between the two groups. Univariate and multivariate logistic regression analyses revealed an increase in HOMA-IR and log_10_ FGF-23 values.

**CONCLUSIONS:**

Insulin resistance, serum FGF-23, and s-KL levels increased in patients compared with healthy individuals. Higher creatinine, proteinuria, and FGF-23 levels were associated with greater insulin resistance. The study highlighted a significant relationship between insulin resistance and FGF-23.

## INTRODUCTION

Insulin mainly targets the liver, skeletal muscle, and fat cells, and regulates glucose metabolism through the insulin receptor. Insulin resistance is defined as at decrease in the sensitivity of targeted organs to circulating insulin. Consequently, the pancreas produces more insulin, which results in hyperinsulinemia. Insulin resistance begins to develop very early in patients with chronic kidney disease (CKD) and becomes more prominent with disease progression disease these patients approach end-stage kidney disease.^1^ Increased visceral fat, accumulation of nitrogenous compounds, metabolic acidosis, vitamin D deficiency, anemia, and physical inactivity lead to the development of insulin resistance in patients with CKD.^2^ Insulin resistance is a risk factor for the progression of renal failure and decreased glomerular filtration rate (GFR). In a study using kidney tissues insulin resistance was demonstrated to be associated with interstitial fibrosis and arteriolosclerosis of renal blood vessels.^3^ Insulin resistance in patients with CKD is also responsible for cardiovascular (CV) diseases that cause mortality.

Fibroblast growth factor-23 (FGF-23) is a hormone that is most frequently synthesized by osteocytes and osteoblasts, less frequently by the spleen and brain, and plays a role in maintaining normal serum phosphate (P) balance. FGF-23 causes renal P excretion by inhibiting sodium-P-2a and sodium-P-2c, which are sodium-dependent P carriers in the proximal tubule.^4^ It also reduces serum 1.25-hydroxy vitamin D3 levels by inhibiting 1-alpha hydroxylase. Klotho (KL) gene is found mainly in the kidneys, parathyroid gland, and choroid plexus.^5^ KL protein reaches the cell membrane from the intracellular endosome, and the extracellular domain of KL is released to circulation as soluble klotho (s-KL). FGF-23 exerts biological activity by binding to fibroblast growth factor receptors in the kidney via an s-KL-dependent pathway. FGF-23 is associated with vascular calcification, inflammation, left ventricular hypertrophy, kidney disease progression, and secondary hyperparathyroidism in patients with CKD.

The association of FGF-23 with atherosclerosis and CV diseases is well-documented; however, only a few previous studies have investigated its association with insulin resistance in pre-dialysis patients with CKD, and the results are contradictory. Therefore, we aimed to investigate the relationship between insulin resistance and FGF-23 levels in patients with non-diabetic pre-dialysis CKD.

## OBJECTIVES

This study aimed to examine the relationship between insulin resistance and FGF-23 and s-KL levels in patients with non-diabetic pre-dialysis CKD.

## METHODS

### Selection of patients

This study was conducted with a total of 283 patients with pre-dialysis CKD, 133 (47%) males and 150 (53%) females, with a mean age of 47.49 ± 9.57 years, who were followed up in the Nephrology Outpatient Clinic. The patients were compared with 80 healthy individuals of a similar age and sex ratios who had no known comorbid diseases or drug use history. Patients with glucose intolerance, fasting blood glucose ≥ 126 mg/dL and/or using antidiabetic drugs, active malignancy, active infection, pregnancy, previous renal transplantation or dialysis history, and who did not want to participate in the study were excluded from the study. After the first urination in the morning, a second urine sample was collected in a container, and the spot urine protein/creatinine ratio (UPCR) was calculated. Body mass index (BMI) was calculated by dividing body weight in kilograms by height in meters squared. The glomerular filtration rate (GFR) was calculated using the Modification of Diet in Renal Disease (MDRD) formula.^6^ The patients were divided into three different CKD stages according to their GFR levels. Those with GFR values between 89-60 mL/min/1.73 ^2^ were evaluated as pre-dialysis stage 3, between 59-30 mL/min/1.73 ^2^ as pre-dialysis stage 4, and those with GFR < 15 mL/min/1.73 ^2^ and those who did not require renal replacement therapy were evaluated as pre-dialysis stage 5. The purpose of the study was explained to all the participants, and the study was approved by the Ethics Committee of the Ankara City Hospital on May 22, 2024 (TABED 1-24-227).

### Determination of laboratory parameters

Venous blood samples were taken from the patients after 8-10 hours of night fasting and 4°C or 10 minutes and stored at -80°C. The levels of creatinine, calcium (Ca), phosphate (P), total cholesterol, triglycerides, and high-density lipoprotein cholesterol (HDL-C) in the collected blood samples were determined using the spectrophotometric method and Beckman Coulter commercial kits in Beckman Coulter AU5800 (Beckman Coulter Inc. CA, USA) autoanalyzer. Low-density lipoprotein cholesterol (LDL-C) levels were calculated according to the formula described by Friedewald et al.^7^ Parathyroid hormone (PTH) levels were determined using a Beckman Coulter Dxl 800 autoanalyzer (Beckman Coulter Inc. CA, USA).1-25-hydroxy (OH) Vitamin(Vit)D_3_ levels were determined using a LIAISON (DiaSorin, MN, USA). Homeostatic model assessment of the insulin resistance (HOMA-IR) value was calculated according to the formula developed by Matthews et al.^8^


$$\text{HOMA}-\text{IR}\;=\;\left[\text{Fasting glucose}\left(\text{in mg/dL}\right)\text{x Fasting insulin}\left(\text{in}\;\text{uIU/mL}\right)\right]/\text{4}0{\text{5}}^{\text{8}}$$

Glycosylated hemoglobin (HbA1c) levels were calculated using high-performance liquid chromatography. Fasting insulin and C-peptide levels were measured via chemiluminescence using a Beckman Coulter DxI800 (Beckman Coulter, Inc. CA, USA) device. Fibroblast growth factor (FGF)-23 and soluble klotho (s-KL) levels were determined using enzyme-linked immunosorbent assay. The measurement range for s-KL was 0.29-18 ng/mL, the measurement sensitivity was 0.15 ng/mL, and the measurement range for FGF-23 was 14.3-895 pg/mL, and the measurement sensitivity was calculated as 8.36 pg/mL.

### Statistical analysis

Data were analyzed using the IBM SPSS software. Conformity to a normal distribution was evaluated using the Kolmogorov-Smirnov test. The Mann-Whitney U test was used to compare data that were not normally distributed among the paired groups. The relationship between groups and categorical variables was examined using Pearson’s Chi-square test, and multiple comparisons were performed using the Bonferroni-corrected Z test. The relationship between non-normally distributed HOMA-IR, clinical characteristics, and laboratory values was examined using Spearman’s rho correlation coefficient. The Kruskal-Wallis H test was used to analyze non-normally distributed variables according to three or more groups. Factors affecting the HOMA-IR levels were examined with the logistic regression model. When all variables were included in the multivariate analysis, in line with the classical method, most results could not be obtained because of inadequate number of observations or the relationship between the independent variables. Therefore, the forward stepwise Wald’s method was used in order to determine the most effective variables. The results of the analyses were presented as frequency (percentage) for categorical variables and as mean ± standard deviation for the quantitative data. The statistical significance level was accepted as 0.05.

## RESULTS

### Patient characteristics

A total of 238 patients with pre-dialysis CKD, 133 males (47%) and 150 females (53%), with a mean age of 47.49 ± 9.57 years were included in the study. The patients were compared with a healthy control group consisting of 80 individuals of similar age and sex. Hypertension was observed in 270 patients (95.4%). Specifically, 49 (17.3%) patients used angiotensin-converting enzyme inhibitors, 137 (48.4%) were on angiotensin II receptor blockers, 119 (42%) were on calcium canal blockers, 70 (24.7%) were on beta-blockers, 22 (7.8%) were on alpha-blockers, 26 (9.2%) were on diuretics, and 30 (10.6%) were on other antihypertensive drugs. According to CKD stages, 150 (53%) patients had stage 3, 84 (29.7%) had stage 4, and 49 (17.3%) had pre-dialysis stage 5. The mean log_10_ FGF-23 was 2.63 ± 0.33 pg/mL, while log_10_ s-KL was 1.28 ± 0.09 ng/mL. The mean HOMA-IR was 3.06 ± 2.92 mg/dL.

When compared to healthy individuals, creatinine, UPCR, PTH, P, log_10_ FGF-23, log_10_ s-KL, triglyceride, HbA1c, C-peptide (all P < 0.001), fasting insulin, and HOMA-IR (P = 0.040) levels were found to be significantly higher in patients,whereas GFR (P < 0.001) was significantly lower. BMI, Ca, and 25-(OH)-Vit D_3_ were similar in both groups (P > 0.005) (**Table 1**).

**Table 1:** Comparison of clinical, demographic and laboratory characteristics of the patient and healthy control group

	Patients (n = 283) Mean ± SD/n(%)	Healthy control group (n = 80) Mean ± SD/n(%)	P value
Age (years)	47.49 ± 9.57	46.21 ± 7.05	0.236*
Male /Female	133 (47%)/ 150 (53%)	40 (50%)/40(50%)	0.635**
BMI (kg/m^2^)	27.63 ± 3.9	26.91 ± 4.49	0.271*
HT	270 (95.4%)		
Use of antihypertensive medications			
ACEinh/ARB	49(17.3%)/ 137(48.4%)		
Calcium channel blocker	119 (42%)		
Beta blocker	70 (24.7%)		
Alpha blocker	22 (7.8%)		
Diuretic	26 (9.2%)		
Others	30 (10.6%)		
CKD			
Stage 3	150 (53%)		
Stage 4	84 (29.7%)		
Pre-dialysis stage 5	49 (17.3%)		
Creatinine (mg/dL)	2.13 ± 0.77	0.84 ± 0.1	< 0.001*
GFR (mL/dk/1.73 m^2^)	33.27 ± 11.49	91.24 ± 6.83	< 0.001*
UPCR (mg/dL)	514.15 ± 540.5	45.15 ± 53.8	< 0.001*
PTH (mg/dL)	107.17 ± 48.32	52.52 ± 23.32	< 0.001*
Ca (mg/dL)	9.35 ± 0.38	9.36 ± 0.4	0.743*
P (mg/dL)	3.76 ± 3.9	2.96 ± 0.62	< 0.001*
25-(OH)-VitD_3_(ng/mL)	21.37 ± 13.26	20.88 ± 10.13	0.981*
Log_10_FGF-23 (pg/mL)	2.63 ± 0.33	2.08 ± 0.28	< 0.001*
Log_10_ s-KL (ng/mL)	1.28 ± 0.09	1.13 ± 0.1	< 0.001*
HbA1c	6.16 ± 1.16	5.59 ± 0.43	< 0.001*
Fasting insulin (pg/mL)	9.46 ± 6.44	7.44 ± 3.48	**0.040**
Total cholesterol (mg/dL)	201 ± 38.57	194.25 ± 41.27	0.106*
Triglyceride (mg/dL)	154.6 ± 61.21	139.06 ± 87.42	< 0.001*
LDL-C (mg/dL)	115.66 ± 38.03	118.65 ± 32.45	0.764*
HDL-C (mg/dL)	54.37 ± 25.24	49.15 ± 14.59	0.209*
C-peptide (ng/mL)	4.58 ± 2.82	2.77 ± 1.14	< 0.001*
HOMA-IR (mg/dL)	3.06 ± 2.92	1.86 ± 1.03	**0.040**

Mann-Whitney U test, Pearson chi-square test, Mean ± standard deviation, Frequency (%).

SD = standard deviation; BMI = body mass index; HT = hypertension; ACEinh = angiotensin-converting enzyme inhibitor; ARB = angiotensin II receptor blocker; CKD = chronic kidney disease; GFR = glomerular filtration rate; FGF-23 = fibroblast growth factor-23; UPCR = urine protein-to-creatinine ratio; PTH = parathyroid hormone; Ca = calcium, P = phosphate; 25(OH)VitD_3_ = 25-hydroxy vitamin D_3_; s-KL = soluble klotho; LDL-C = low-density lipoprotein cholesterol; HDL-C = high-density lipoprotein cholesterol; HbA1c = glycosylated hemoglobin; HOMA-IR = homeostasis model assessment of insulin resistance.

### Mean insulin resistance values

Compared to those with HOMA-IR ≤ 3.06, stage 5 CKD (P = 0.002), BMI, UPCR (P < 0.001), creatinine (P = 0.002), PTH (P = 0.025), and log_10_ FGF-23 (P = 0.003) were significantly higher in those with HOMA-IR > 3.06, whereas GFR, Ca, P, and 25(OH)VitD_3_ values were significantly lower (P < 0.001). Both groups had similar s-KL levels (**Table 2**).

**Table 2:** Comparison of clinical and laboratory features according to mean homeostasis model assessment of insulin resistance levels

	HOMA-IR = 3.06 (n = 209)	HOMA-IR > 3.06 (n = 74)	P value
BMI (kg/m^2^)	29.82 ± 5.14	26.85 ± 3.01	< 0.001*
CKD			
Stage 3	123 (58.9%)^a^	27 (36.5%)^b^	
Stage 4	57 (27.3%)^a^	27 (36.5%)^a^	0.002**
Pre-dialysis stage 5	29 (13.9%)^a^	20 (27%)^b^	
Creatinine (mg/dL)	2.01 ± 0.66	2.47 ± 0.94	0.002*
GFR (mL/dk/1.73 m^2^)	34.52 ± 11.35	29.76 ± 11.23	< 0.001*
UPCR (mg/dL)	352.49 ± 373.03	970.74 ± 667.78	< 0.001*
PTH (mg/dL)	103.54 ± 46.02	117.43 ± 53.27	0.025*
Ca (mg/dL)	9.37 ± 0.41	9.29 ± 0.26	< 0.001*
P (mg/dL)	3.05 ± 0.61	2.71 ± 0.6	< 0.001*
25-(OH)-VitD_3_(ng/mL)	24.2 ± 14.1	13.83 ± 6.11	< 0.001*
Log_10_ FGF-23 (pg/mL)	2.6 ± 0.31	2.69 ± 0.36	0.003*
Log_10_s-KL (ng/mL)	1.29 ± 0.09	1.27 ± 0.11	0.075*

Mann -Whitney U test, Pearson chi-square test, a-b: There is no difference between groups with the same letter (Z test with Bonferroni correction), Mean ± standard deviation, Frequency (%).

BMI = body mass index; CKD = chronic kidney disease; GFR = glomerular filtration rate; UPCR = urine protein-to-creatinine ratio; PTH = parathyroid hormone; Ca = calcium, P = phosphate; 25(OH)VitD_3_ = 25-hydroxy vitamin D_3_; s-KL = soluble klotho; HOMA-IR = homeostasis model assessment of insulin resistance; FGF-23 = fibroblast growth factor-23.

### The relationship of insulin resistance with FGF-23 and s-KL

**Figure 1:**
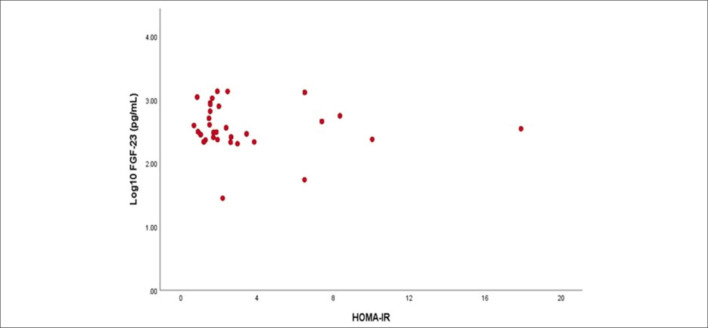
Relationship between homeostasis model assessment of insulin resistance and fibroblast growth factor-23.

**Figure 2:**
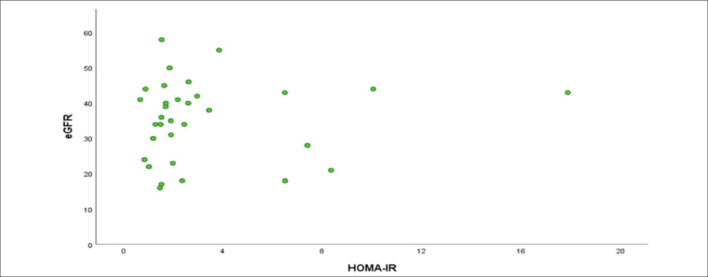
Relationship between homeostasis model assessment of insulin resistance and glomerular filtration rate.

**Figure 3:**
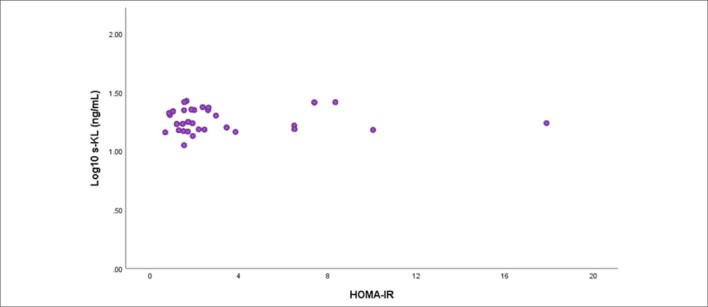
Relationship between homeostasis model assessment of insulin resistance and soluble klotho.

In the univariate logistic regression analysis, as BMI increased, HOMA-IR levels increased 1.212 times OR = 1.212, 95% CI 1.128–1.302). As the log_10_ FGF-23 value increased, the HOMA-IR values increased 2.425 times (OR = 2.425, 95% CI 1.032–5.698) (**Figure 1**). An increase in the UPCR value led to an increase of 1.002 times in the HOMA-IR values (OR = 1.002, 95%CI 1.002–1.003). As PTH levels increased, HOMA-IR levels increased by 1.006 times (OR = 1.006, 95% CI 1.000–1.011). As the GFR value increased, a decrease of 0.962 times was observed in the HOMA-IR levels (OR = 0.962, 95% CI 0.939–0.987) (**Figure 2**). As the P- value increased, HOMA-IR levels decreased by 0.414 times (OR = 0.414, 95% CI 0.266–0.645). As the 25(OH)VitD_3_ value increased, a decrease of 0.899 times was observed in the HOMA-IR levels (OR = 0.899, 95% CI 0.865–0.933). No relationship was found between HOMA-IR and s-KL levels (P > 0.05) (**Figure 3**).

According to the multivariate logistic regression analysis using the forward stepwise Wald’s method, as the BMI increased, HOMA-IR levels increased 2.933 times (OR = 2.933, 95% CI 2.073–4.150). As the log_10_ FGF-23 value increased, this led to a decrease of 0.002 times in HOMA-IR levels (OR = 0.002, 95%CI = 0.000–0.033). As the PTH level increased, a decrease of 0.963 times was observed in the HOMA-IR levels (OR = 0.963, 95% CI 0.947–0.979). As the Ca level increased, the HOMA-IR levels decreased by 0.053 (OR = 0.053, 95% CI 0.015–0.183). As the P-value increased, a decrease of 17.239 times was observed in HOMA-IR levels (OR = 17.239, 95%CI 2.848–104.366). As the 25(OH)VitD_3_ increased, the HOMA-IR levels decreased by 0.836 (OR = 0.836, 95%CI 0.784–0.891). The common effects of the other parameters on the HOMA-IR levels were not statistically significant (P > 0.05) (**Table 3**).

**Table 3:** Factors independently affecting homeostasis model assessment of insulin resistance

	Univariate		Multivariate^fwald^	
	OR (%95 CI)	P	OR (%95 CI)	P
BMI (kg/m^2^)	1.212 (1.128 - 1.302)	**< 0.001**	2.933 (2.073 - 4.15)	**< 0.001**
GFR (mL/dk/1.73 m^2^)	0.962 (0.939 - 0.987)	**0.003**		
UPCR (mg/dL)	1.002 (1.002 - 1.003)	**< 0.001**		
PTH (mg/dL)	1.006 (1 - 1.011)	**0.035**	0.963 (0.947 - 0.979)	**< 0.001**
Ca (mg/dL)	0.593 (0.299 - 1.176)	0.135	0.053 (0.015 - 0.183)	**< 0.001**
P (mg/dL)	0.414 (0.266 - 0.645)	**< 0.001**	17.239 (2.848 - 104.366)	**0.002**
25-(OH)-VitD_3_ (ng/mL)	0.899 (0.865 - 0.933)	**< 0.001**	0.836 (0.784 - 0.891)	**< 0.001**
Log_10_FGF-23 (pg/mL)	2.425 (1.032 - 5.698)	**0.042**	**0.002 (0 - 0.033)**	**< 0.001**
Log_10_ s-KL (ng/mL)	0.084 (0.005 - 1.412)	0.085		

OR = Odds ratio, CI = Confidence interval, fwald = Forward Wald Method, Accuracy = 0.963; BMI = body mass index; CKD = chronic kidney disease; GFR = glomerular filtration rate; UPCR = urine protein-to-creatinine ratio; PTH = parathyroid hormone; Ca = calcium, P = phosphate; 25(OH)VitD_3_ = 25-hydroxy vitamin D_3_; s-KL = soluble klotho; HOMA-IR = homeostasis model assessment of insulin resistance; FGF-23 = fibroblast growth factor-23.

## DISCUSSION

In this study, elevated insulin resistance development and elevated serum FGF-23 and s-KL levels were observed in patients with CKD in comparison to healthy individuals. Higher creatinine levels, proteinuria, and serum FGF-23 levels were observed in patients with high insulin resistance than in those with lower levels. A significant relationship, independent of serum P levels, was observed between insulin resistance and FGF-23 levels.

Insulin resistance is characterized by a deteriorated physiological response of peripheral tissues to the metabolic effects of insulin; and occurs due to a decrease in insulin receptor expression in tissues that play a role in energy homeostasis. Insulin resistance in patients with CKD is a metabolic feature and an independent determinant of mortality in the early and late stages of CKD. In this study, increased insulin resistance development identified with HOMA-IR was determined in patients with CKD compared to healthy individuals. It was also determined that as renal function disorders and proteinuria progressed, insulin resistance increased. Arroyo et al. reported that insulin resistance determined by HOMA-IR was high in patients with stage 3-4 CKD.^9^ Duong et al. reported in their study that HOMA-IR was high among patients who received dialysis and that it correlated with inflammatory markers.^10^ Du et al. reported the occurrence of insulin resistance starting from the early CKD stages.^11^ In contrast to these aforementioned studies, Park et al., in their large-scale study including 17.157 individuals, reported that GFR decrease was not associated with the increase in insulin resistance.^12^ Chen et al. reported in their study that there was a significant relationship between insulin resistance and GFR in patients with CKD. However, this relationship disappeared after conductng a multivariate analyses, which included BMI as correction factor.^13^

FGF-23 is a hormone synthesized by osteocytes and osteoblasts and plays a role in controlling serum P levels. s-KL is synthesized by the epithelial cells of the kidney tubules, most frequently from the distal tubule. When FGF-23 binds to s-KL in the kidneys, fibroblast growth factor receptors become activated, inhibiting renal P reabsorption and leading to a decrease in serum 1.25(OH)(Vit)D_3_ by inhibiting the 1-alpha-hydroxylase enzyme. As the P load per nephron increases in patients with CKD, serum FGF-23 levels increase to counterbalance this load. In the present study, serum FGF-23 and s-KL levels were observed to be higher in patients with CKD than in healthy individuals. Lima et al. reported that there was an increase in serum FGF-23 levels in patients with CKD starting from the early stages.^14^ Manou et al. reported in their study that as the renal failure stage progressed in patients with CKD, the increase in serum FGF-23 levels was accompanied by a decrease in the coreceptor s-KL levels.^15^ Shou et al. reported that increased serum FGF-23 levels in patients with advanced-stage CKD were accompanied by an increase in serum PTH levels, along with a decrease in 1. 1.25(OH)(Vit)D_3_ levels.^16^

Kutluturk et al. conducted a study including 46 obese children and adults in which they observed that serum insulin and glucose levels increased, while FGF-23 and s-KL decreased. They identified an inverse relationship between insulin resistance and FGF-23. They claimed that insulin resistance-related hyperinsulinism and/or low 1.25(OH)(Vit)D_3_ levels may lead to a decrease in FGF-23 generation and serum levels. This study focused on adolescents with a mean age of 14 years and no renal failure,^17^ Wojcik et al. reported an inverse correlation between insulin resistance and FGF-23.^18^ In another study they conducted on obese adolescents with insulin resistance and without renal failure, they reported lower FGF-23 levels when compared to obese controls who did not have insulin resistance. They claimed that lower insulin resistance was correlated with higher FGF-23 levels.^19^ Hanks et al. reported that insulin resistance determined by HOMA-IR was associated with FGF-23, especially in those who had normal renal functions, but that this relationship was not observed in patients with CKD. They claimed that FGF-23 in circulation modulated the bonding of s-KL and thus indirectly affected insulin signal.^20^ Holecki et al. reported in their study on 3115 elderly individuals that there was no relationship between insulin resistance and FGF-23.^21^ Mirza et al. reported in The Prospective Investigation of the Vasculature in Uppsala Seniors Cohort (PIVUS) and The Osteoporotic Fractures in Men Study Cohort (MrOS) studies that one standard deviation increase in FGF-23 was associated with 8% and 12% higher insulin and HOMA-IR values. However, in the multivariate analysis, they reported no correlation between insulin resistance and FGF-23.^22^ Unlike these studies, in our study, higher serum FGF-23 levels and lower serum P levels were observed in patients with high insulin resistance. No difference was observed in the s-KL levels between patients with high and low insulin resistance. Winther et al. reported that hyperinsulinemia increased serum FGF-23 levels in patients with type 2 DM.^23^ Marchelek-Mysliwiec reported that FGF-23 was a significant determinant of insulin resistance in patients with CKD.^24^ Fernandez-Real et al. determined the relationship between HOMA-IR and FGF-23 in their study on 314 individuals with renal failure. They reported that HOMA-IR and FGF-23 levels decreased in 10 males after losing about 20 kg and that insulin resistance affected FGF-23 levels.^25^ Garland et al. reported higher FGF-23 levels in those with high insulin resistance in their study on 72 patients with stage 3-5 CKD. They reported a relationship between insulin resistance and P balance, and found a significant and independent relationship between insulin resistance and FGF-23. They argued that insulin resistance contributes to the deterioration of renal P homeostasis in patients with CKD and that this could be determined clinically with increased FGF-23 levels.^26^ In the present study, while a significant relationship was observed between insulin resistance and FGF-23, an inverse correlation with P was found. No relationship was observed between insulin resistance and s-KL levels. This suggests that the relationship between insulin resistance and FGF-23 levels in patients with CKD may result from mechanisms other than P metabolism. However further studies are required to confirm this hypothesis.

The varying results of these previous studies can be attributed to various reasons. FGF-23 was divided into two kits intact and C-terminal. However, the FGF-23 kits used in these studies may have been different. It is thought that C-terminal FGF-23 better reflects the biologically functional FGF-23 molecule that reduces the reabsorption of P in kidney tubules.^27^ In their study on 81patients with CKD younger than 25 years, Yasin et al. reported that FGF-23 levels varied depending on age.^28^ The type of FGF-23 kits used, the ages of the patients included, and their growth status may have yielded different results. The demonstration of the effect of insulin on FGF-23 release through the hyperinsulinemic-euglycemic clamp technique^23^ suggests that high insulin levels are required for FGF-23 synthesis. The insulin levels in these aforementioned studies could have been too low to cause this effect.

Certain limitations affect the results of our study. First, as the study was conducted using a cross-sectional design, the temporal results of the relationship between insulin resistance and FGF-23 in patients with CKD were not examined. Second, instead of the hyperinsulinemic-euglycemic technique, which is the gold standard method for determining insulin resistance, the HOMA-IR formula was used. Third, the FGF-23 values, which are FGF subgroups that regulate glucose metabolism in patients with CKD,^29^ were not included in the study. Fourth, although the relationship between FGF-23, insulin resistance, and inflammatory parameters, such as interleukin, vascular cell adhesion molecule, and tumor necrosis factor, is known, these markers have not been studied. Fifth, although the anti-phosphaturic activities of insulin and FGF-23 are known, their effects on serum and urine levels have not been investigated.

## CONCLUSION

Increased insulin resistance development and increased serum FGF-23 and s-KL levels were observed in patients with pre-dialysis CKD when compared with healthy individuals. Higher insulin resistance was observed in patients with stage 5 CKD than in those with stage 3 CKD. More renal function disorders and higher serum FGF-23 levels were observed in patients with high insulin resistance. Although a significant relationship was found between insulin resistance and FGF-23 levels, an inverse correlation was found with P. As few studies have been conducted in this regard, further multicenter studies with larger number of patients are needed.
